# Immunologic Signatures across Molecular Subtypes and Potential Biomarkers for Sub-Stratification in Endometrial Cancer

**DOI:** 10.3390/ijms24021791

**Published:** 2023-01-16

**Authors:** Fang Jiang, Shiyang Jiang, Dongyan Cao, Mingyi Mao, Yang Xiang

**Affiliations:** Department of Obstetrics and Gynecology, Peking Union Medical College Hospital, Chinese Academy of Medical Sciences & Peking Union Medical College, National Clinical Research Center for Obstetric & Gynecologic Diseases, Beijing 100730, China

**Keywords:** endometrial cancer, molecular subtype, next-generation sequencing, immunologic signature, risk stratification, prognostic marker

## Abstract

Current molecular classification approaches for endometrial cancer (EC) often employ multiple testing platforms. Some subtypes still lack univocal prognostic significance, highlighting the need for risk sub-stratification. The tumor immune microenvironment (TIME) is associated with tumor progression and prognosis. We sought to investigate the feasibility of classifying EC via DNA sequencing and interrogate immunologic signatures and prognostic markers across and within subtypes, respectively. Formalin-fixed paraffin-embedding (FFPE) samples from 50 EC patients underwent targeted DNA and RNA sequencing, and multiplex immunofluorescence assay for TIME. DNA sequencing classified 10%, 20%, 52%, and 18% of patients into the subtype of *POLE*-mutant, microsatellite instability-high (MSI-H), *TP53*-wt, and *TP53*-mutant. *POLE*-mutant tumors expressed the highest T-effector and IFN-γ signature and the lowest innate anti-PD-1 resistance signature among subtypes. *TP53*-wt revealed a converse enrichment trend for these immunologic signatures. Survival analyses using the Cancer Genome Atlas Uterine Corpus Endometrial Carcinoma (TCGA-UCEC) dataset identified associations of *CCR5* (hazard ratio (HR) = 0.71, *p* = 0.035), *TNFRSF14* (HR = 0.58, *p* = 0.028), and *IL-10* (HR = 2.5, *p* = 0.012) with overall survival within MSI-H, *TP53*-mutant, and *TP53*-wt subtype, respectively. A TIME comparison between the sub-stratified subgroups of our cohort revealed upregulated tumor infiltration of immune cells in the low-risk subgroups. Our study demonstrates that targeted DNA sequencing is an effective one-stop strategy to classify EC. Immunomodulatory genes may serve as prognostic markers within subtypes.

## 1. Introduction

Uterine corpus cancer is the second most common gynecological cancer worldwide [1] and its incidence is rapidly rising in China [2]. Endometrial cancer (EC) comprises most uterine cancer cases. As a highly heterogeneous entity, EC can be divided into various histologic subtypes: mainly endometrioid endometrial cancer (EEC), serous endometrial cancer (SEC), and clear cell endometrial cancer (CCEC). Currently, pathologic evaluation is routinely used in clinical practice to predict prognosis and guide the management of EC patients.

With the expanded understanding of the EC genome, extensive investigations have demonstrated the potential clinical utility of molecular subtyping EC for risk stratification and selecting optimal treatment. Using array and sequencing-based technologies, the Cancer Genome Atlas (TCGA) research network first divided EECs and SECs into four molecular subtypes with prognostic significance: the POLE/ultramutated subtype exhibited the most favorable prognosis; the microsatellite instability (MSI)/hypermutated and copy-number low subtypes showed intermediate prognosis; the copy-number high subtype, also characterized by a low mutation burden and a high *TP53* mutation frequency, had poor prognosis [3]. Afterward, to broaden the utility of TCGA classification, other research groups used immunohistochemical (IHC) assessment of mismatch repair (MMR) proteins and p53 as surrogates for testing MSI and somatic copy-number alterations (SCNA). The Translational Research in Post-Operative Radiation Therapy in EC (TransPORTEC) system defines four subtypes: p53 abnormal, MSI, *POLE*-mutant, or no specific molecular profile (NSMP) [4,5]. The ProMisE (Proactive Molecular Risk Classifier for Endometrial Cancer) system classifies patients in the light of testing for aberrations in the sequence of MMR deficiency (dMMR), *POLE* mutation, and p53 abnormality [6]. Despite showing promising values in risk stratification, these molecular classifiers integrate results from multiple testing platforms that are labor-intensive. A highly professional and multi-disciplinary team is also required to interpret the results. These drawbacks will largely confine the clinical application of these classification schemes. Developing an easy-handling and clinically feasible approach for molecular subtyping of EC remains an unmet need.

Although incorporating molecular subtypes into risk stratification can limit the current under- and overtreatment of EC patients, some molecular subtypes still lack a univocal prognostic significance, especially the NSMP group, the prognosis of which is largely affected by clinicopathological features of tumors [4,5]. The intra-subtype heterogeneity suggests that further stratifying subtypes based on prognostic factors may improve the current molecular classification [7].

Mounting evidence reveals the role of the immune system in the progression and prognosis of various tumors, including EC. The favorable prognosis of *POLE*-mutant and dMMR subtypes has been suggested to correlate with increased density of CD8+ tumor-infiltrating lymphocytes (TILs) and higher PD-1expression [8,9]. van Gool et al. showed that the upregulated cytotoxic T cells in *POLE*-mutant tumors were accompanied by enriched tumor-infiltrating T-cell gene expression signature and increased expression of T-cell cytotoxic differentiation and effector markers [10]. Talhouk et al. characterized the immune microenvironment across the four molecular subtypes of EC and found profound variation in immune response across and within subtypes [11]. Collectively, these results suggest the potential for using immunologic signatures to sub-stratify patients within subtypes.

Herein, we classified women with EC using a one-stop strategy based on targeted DNA sequencing and interrogated immunologic signatures across subtypes, including transcriptome of immunomodulatory genes and tumor immune microenvironment (TIME) landscape. We also investigated the prognostic value of immunomodulatory genes within individual subtypes using the TCGA cohort.

## 2. Results

### 2.1. Characteristics of Patients

A total of 50 women with EC were included in the study, with a median age of 57 years. Most patients presented with stage I or II (62%), endometrioid (86%), and low-grade (G1 or G2, 70%) tumors. Three and four patients had serous and clear cell histology, respectively (Table 1). The cohort had a postoperative follow-up of 10 to 14 months. Up to the last follow-up date, the progression-free survival (PFS) rate was 94%. Three (6%) out of fifty patients developed recurrent or progressive disease.

### 2.2. Molecular Subtyping of EC via a One-Stop Next-Generation Sequencing (NGS) Strategy

**Figure 1:**
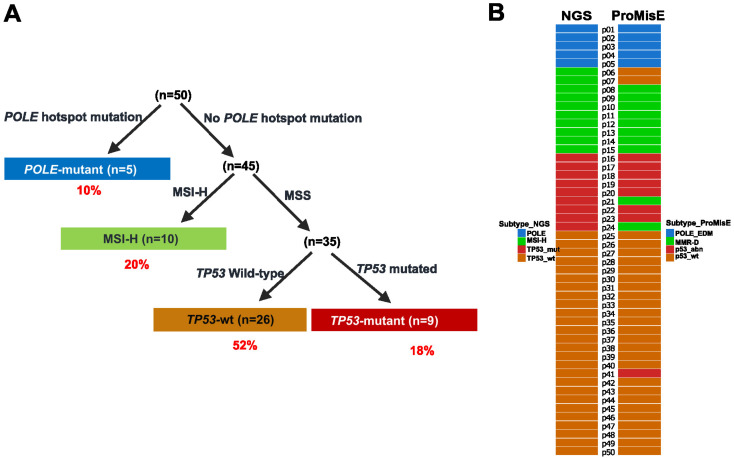
NGS one-stop molecular subtyping of 50 patients with endometrial cancer: (**A**) Flowchart of molecular subtyping of patients by NGS; (**B**) Comparison of classification between NGS strategy and IHC-based ProMisE approach. Abbreviation: MSI-H: microsatellite instability high; MSS: microsatellite stable; MMR-D: DNA mismatch repair deficient;.

Patients were subtyped based on the genomic signatures identified by DNA sequencing in the order of *POLE* hotspot mutation, MSI-high (MSI-H), and *TP53* mutation. Eventually, five (10%), ten (20%), twenty-six (52%), and nine (18%) patients were categorized into the subtype of *POLE*-mutant, MSI-H, *TP53*-wt, and *TP53*-mutant, respectively (Figure 1A). The *TP53*-mutant subtype showed lower estrogen receptor (ER) and progesterone receptor (RP) positive rates than other subtypes (*p* = 0.006, *p* = 0.002, Table 1). A higher frequency (100%) of lymphovascular space invasion was observed in the *POLE*-mutant subtype (*p* = 0.048). *TP53*-wt tumors displayed lower Ki67 expression than other subtypes (*p* = 0.001).

We also performed IHC for MMR and p53 proteins and classified patients according to the ProMisE system. Figure 1B illustrates the comparison of classification between the two approaches. Of the 50 patients, only 5 had discordant subtypes, resulting in a concordance of 90%. Specifically, patients p06 and p07 were identified with an MSI-H status by NGS but with a proficient MMR (pMMR) and p53-wt status by IHC, therefore were classified into the subtype of MSI-H by NGS and p53-wt by ProMisE. Patients p21 and p24 were determined as microsatellite stable (MSS) and *TP53*-mutant by NGS but deficient MMR (MMR-D) by IHC, resulting in the discordant subtyping between NGS (*TP53*-mutant) and ProMisE (MMR-D). Patient 41 was classified as *TP53*-wt by NGS and p53-abn by ProMisE.

### 2.3. Distinctive Genomic Profiles among Different Molecular Subtypes

**Figure 2:**
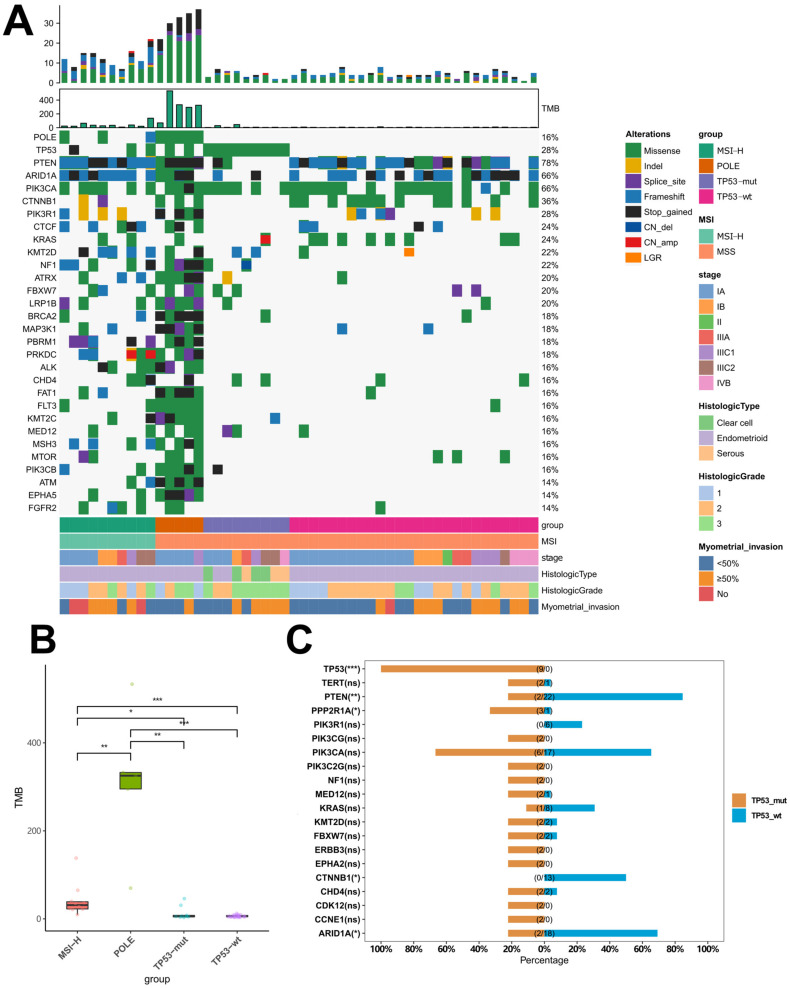
Genomic profiles of patients with different molecular subtypes of endometrial cancer: (**A**) Somatic mutation profile of patients (*n* = 50). Different colors on the oncoprint represent the mutation types. Only the genes with a mutation rate of ≥10% were included in the oncoprint; (**B**) Comparison of TMB among four different subtypes; (**C**) Comparison of somatic gene mutation rates between *TP53*-mutant and wildtype subtypes. Asterisks denote statistical significance; *p* < 0.05 (*); *p* < 0.01 (**); *p* < 0.005 (***); ns: not statistically significant.

*PTEN* (78%), *ARID1A* (66%), and *PIK3CA* (66%) mutated the most frequently in our cohort (Figure 2A). *PIK3CA* mutation occurred commonly across four subtypes (≥60%), while *PTEN* and *ARID1A* mutations were not that common in the *TP53*-mutant subtype (22.2%, Figure S1). *POLE*-mutant tumors gained a massive amount of mutations conferring an extremely high median tumor mutational burden (TMB) (325.40 mutations/Mb). MSI-H subtype ranked second (31.35 mutations/Mb). *TP53*-wt and *TP53*-mutant subtypes had comparable low TMB (5.56 vs. 5.96 mutations/Mb) (Figure 2B). Next, we compared the mutation profile between *TP53*-mutant and -wt subtypes, and found the former harbored more mutations in *PPP2R1A* (33.3% vs. 3.8%, *p* = 0.044) and less common mutations in *PTEN* (22.2% vs. 84.6%, *p* < 0.01), *ARID1A* (22.2% vs. 69.2%, *p* = 0.022, *CTNNB1* (0% vs. 50%, *p* = 0.013) (Figure 2C).

### 2.4. Differential Immunologic Signatures across Four Subtypes

**Figure 3:**
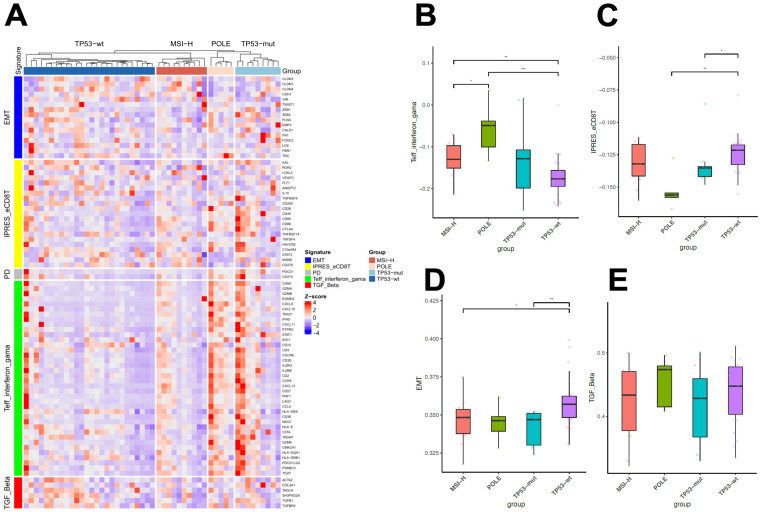
Expression of immunologic genes across four different molecular subtypes of endometrial cancer: (**A**) Heatmap showing expression of 83 T-cell inflamed genes according to endometrial cancer molecular subtype. Gene-level expression values were computed as transcripts per million (TPM) and were normalized to Z-scores before clustering. Genes were arranged according to signatures; (**B**–**E**) Comparison of single sample Gene Set Variance Analysis (ssGSVA) scores of gene signatures across subtypes, including signatures of T-effector and interferon-gama (Teff_interferon_gama) (**B**), innate anti-PD-1 resistance and CD8+ T cell exhaustion (IPRES_eCD8T) (**C**), epithelial-mesenchymal transition (EMT) (**D**) and TGF-Beta (**E**). Asterisks denote statistical significance; *p* < 0.05 (*); *p* < 0.01 (**); *p* < 0.005 (***).

Next, we interrogated the transcriptome profile of 83 immunologic genes among four EC molecular subtypes (Figure 3A). Differential expression levels were observed across subtypes for numerous genes related to T-effector and interferon-gamma (IFN-γ) gene signature and innate anti-PD-1 resistance and CD8+ T cell exhaustion (IPRES_eCD8T) signature (Table S1). When comparing signature enrichment based on single-sample Gene Set Variation Analysis (ssGSVA) scores, we found *POLE*-mutant tumors showed the highest enrichment for the signature of T-effector and IFN-γ, and the MSI-H subtype ranked second, followed by *TP53*-wt. *TP53*-mutant tumors did not show significantly differential enrichment for this signature vs. other subtypes (Figure 3B). Conversely, the enrichment score for the IPRES_eCD8T signature was lower in the *POLE*-mutant vs. *TP53*-wt tumors (*p* < 0.01), and the latter showed a higher score than the *TP53*-mutant subtype (*p* = 0.025) (Figure 3C). We also observed higher enrichment for the epithelial-mesenchymal transition (EMT) signature in *TP53*-wt tumors than in *TP53*-mutant (*p* < 0.01) and MSI-H subtypes (*p* = 0.014) (Figure 3D). The transforming growth factor-Beta (TGF-Beta) signature was not enriched differentially across four subtypes (Figure 3E).

We also investigated the TIME landscape among different subtypes by assessing the tumor-infiltrating immune cells. A differential rate of CD8+ TILs was observed across subtypes in the tumor epithelial compartment (*p* = 0.029, Table S2). Specifically, *POLE*-mutant and *TP53*-mutant tumors showed higher rates of epithelial CD8+TILs than *TP5*3-wt tumors (Figure S2A). In the stromal compartment, *POLE*-mutant (*p* < 0.01, *p* < 0.01) and MSI-H (*p* < 0.05, *p* < 0.01) subtypes expressed both higher densities and rates of PD-L1+ marker than *TP53*-wt tumors, and a higher rate of CD8+TILs was observed in *POLE*-mutant (*p* < 0.05) and *TP53*-mutant (*p* < 0.01) tumors than in *TP5*3-wt subtype (Figure S2B). Other immune markers were generally expressed comparably across different molecular subtypes (Table S2).

### 2.5. Prognostic Value of Immunomodulatory Genes

**Figure 4:**
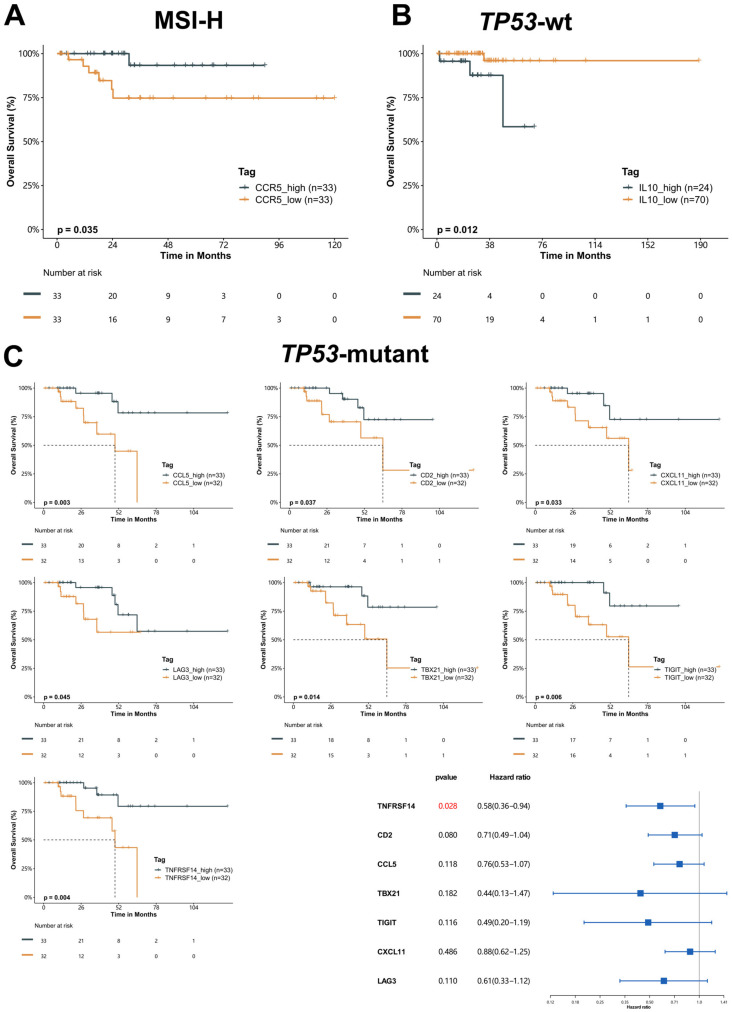
Survival-associated genes within molecular subtypes of endometrial cancer: (**A**) Kaplan-Meier (K-M) curve of patients with MSI-H subtype stratified by the expression of CCR5; (**B**) K-M curve of patients with TP53-wt subtype stratified by the expression of IL-10; (**C**) K-M curves of patients with TP53-mutant subtype stratified by the expression of CCR5, CD2, CXCL11, LAG3, TBX21, TNFRSF14, and TIGIT as well as forest plot illustrating the results from the multivariate Cox regression analysis. The median expression level was selected as the cut-off for all genes except for IL-10, for which the cut-off was the upper quartile.

Despite differential expression profiles of immunomodulatory genes across subtypes, we also observed heterogeneous expression patterns among tumors within the same subtype (Figure 3A), suggesting the potential of further stratifying patients using these genes. Therefore, we retrieved mRNA expression data of the 83 genes covered in the present study and clinical information from 232 EC patients, whose molecular subtypes had been released [3], from the TCGA Uterine Corpus Endometrial Carcinoma- (TCGA-UCEC) database. First, we analyzed the association of individual gene expression with prognosis and identified 24 genes significantly associated with overall survival (OS) (Table S3, *p* < 0.05). Subsequently, we assessed the prognostic value of these candidate genes within each molecular subtype, except for *POLE*-mutant tumors, which invariably exhibit the most favorable prognosis regardless of clinicopathological features [7]. Using the median expression value of *CCR5*, we stratified patients with MSI-H tumors into two subgroups with differential OS (hazard ratio (HR) = 0.71, *p* = 0.035) (Figure 4A). Conversely, a higher expression of *IL10* yielded a shorter median OS within the *TP53*-wt subtype (HR = 2.5, *p* = 0.012, Figure 4B). Within *TP53*-mutant subtype, we found higher expression levels of *CCR5* (*p* = 0.003), *CD2* (*p* = 0.037), *CXCL11* (*p* = 0.033), *LAG3* (*p* = 0.045), *TBX21* (*p* = 0.014), *TNFRSF14* (*p* = 0.004), and *TIGIT* (*p* = 0.006) were associated with more favorable survivals (Figure 4C). Multivariate analysis confirmed *TNFRSF14* as an independent prognostic factor in patients with *TP53*-mutant EC (HR = 0.58 (0.36−0.94), *p* = 0.028).

**Figure 5:**
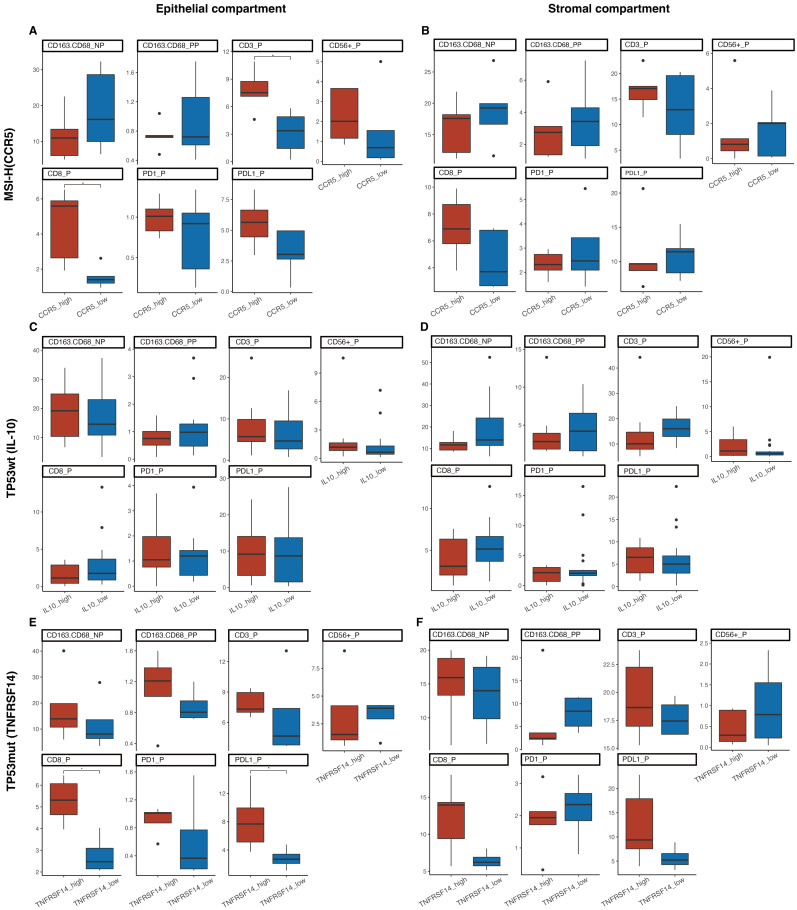
Comparison of tumor immune microenvironment (TIME) landscape between the subsets within molecular subtypes of endometrial cancer sub-stratified by expression of immune-related genes. Intraepithelial and intrastromal positive rates of immune markers were compared. MSI-H subtype was sub-stratified by the expression of *CCR5* using median expression level as the cut-off (**A**,**B**). *TP53*-wt subtype was sub-stratified by the expression of *IL10* using upper quartile expression level as the cut-off (**C**,**D**). *TP53*-mutant subtype was sub-stratified by the expression of *TNFRSF14* using median expression level as the cut-off (**E**,**F**). Asterisks denote statistical significance; *p* < 0.05 (*).

Next, we sub-stratified the patients of our cohort using the same cut-offs of the above-mentioned prognostic genes and compared the TIME landscape between subgroups within each molecular subtype (Table S4). The *CCR5*-high subset of MSI-H tumors revealed higher positive rates of intraepithelial CD3+ and CD8+ TILs compared with the *CCR5*-low subset (Figure 5A). The *TNFRSF14*-high subset of *TP53*-mutant tumors expressed higher positive rates of intraepithelial PD-L1+ markers and CD8+ TILs than the *TNFRSF14*-high subset (Figure 5E). TIME signatures were generally comparable in the stromal compartment between subsets of MSI-H and *TP53*-mutant tumors (Figure 5B,F). Moreover, we did not observe significantly differential TIME signatures in the epithelial compartment between the two subsets of *TP53*-wt tumors sub-stratified by *IL10* expression (Figure 5C). While the positive rate of CD163−CD68+ cells (putative M1 macrophages) in the stromal area tended to be lower in the *IL10*-high subset (*p* = 0.078, Table S4, Figure 5D)).

Notably, one patient with stage IIIC1, G1, and MSI-H EC developed disease progression during the postoperative treatment with radiotherapy, chemotherapy, and PD-1 inhibitor. The patient was sub-stratified as *CCR5*-low, suggestive of an inferior prognosis. In the *TP53*-wt group, one patient with stage IIIC1 G1 EC refused adjuvant therapy, and systemic recurrence was noted six months after surgery. The patient was also sub-stratified into a high-risk subset featured by *IL10*-high. In the *TP53*-mutant group, one patient with high-grade serous carcinoma staged as IVB experienced intraperitoneal recurrence two months after finishing the standard adjuvant therapy. The patient was characterized as *TNFRF14*-low, indicating a poor prognosis.

## 3. Discussion

In this study, we performed molecular subtyping of 50 EC patients using targeted DNA sequencing and discovered differential immunologic gene signatures and TIME across varied subtypes. In addition, we also identified *CCR5*, *TNFRSF14*, and *IL-10* that are associated with OS within MSI-H, *TP53*-mutant, and *TP53*-wt subtypes, respectively.

Our study classified 43 EECs, 3 SECs, and 4 CCECs into four molecular subtypes through a one-stop NGS-based strategy. Compared with TransPORTEC and ProMisE systems, our approach, which can be conducted largely in an automatic manner, is more labor-saving. It also relies less on experienced pathologists, which may introduce inter-observer variability. In our study, four patients show discordant results between IHC and NGS results for MSI. Two MSI-H/pMMR cases can be caused by inactive mutant proteins that remain detectable by IHC. Due to functional redundancy, cases with isolated loss of MMR protein may still have MMR function, which may explain the MSS/dMMR results in two patients [12,13]. In these two scenarios, NGS outperforms IHC since it reflects the actual status of MMR function. Moreover, because it was found in 92% of copy number-high tumors, the *TP53* mutation is considered a surrogate for copy number-high subtyping [3]. Given the discordance of ~8% between mutation and protein status of TP53, the routinely used IHC would misclassify ~15% copy number-high tumors into p53-wt/NSMP subtype [14,15]. In this regard, our NGS strategy would reduce such misclassifications. However, it should be noted that the current cost of NGS may prevent the implementation of our strategy. However, with the rapid development of high throughput sequencing, the cost per sample will ultimately reduce to an affordable range. In addition to molecular subtyping, NGS can provide information that may guide the therapeutic decision. This advantage will promote the utility of the NGS strategy in the clinic.

We present the first study comprehensively investigating the immunomodulatory transcriptome signatures across four molecular subtypes of EC. Our study showed an upregulation of T-effector and IFN-γ signature in *POLE*-mutant and MSI-H tumors and downregulation of IPRES signature in *POLE*-mutant tumors. The gene signature of T-effector and IFN-γ, indicative of a T-cell–activated tumor environment, has been associated with response to PD-1 blockade [16]. Contrastingly, the IPRES signature defines a set of genes related to immune-suppressive cytokines, EMT transcription factors, and pro-angiogenic factors and has been associated with innate resistance to PD-1 blockade across multiple cancers [17]. Moreover, the *TP53*-wt subtype expressed a higher level EMT signature. Despite being a major inductor of EMT, TGF-β related gene expression was comparable among different subtypes. This discordance may be due to the relatively limited sample size of our cohort, given that the *TP53*-wt tumors showed a numerically higher median level of TGF-β signature than *TP53*-mutant and MSI-H tumors (Figure 3E). Another possible explanation is that the EMT process in the *TP53*-wt tumors can be induced by activation of other signaling pathways such as the Wnt/β-catenin pathway [18].

Our study also depicts differential TIME landscapes across four subtypes of EC. Higher rates and densities of PD-L1+ cells were found in stromal areas of *POLE*-mutant and MSI-H tumors, and higher rates of CD8+TILs were seen in both tumoral and stromal areas of *POLE*-mutant tumors. We also found lower rates of both tumoral and stromal CD8+TILs present in *TP53*-wt than in *TP53*-mutant tumors. Consistently, studies have described higher numbers of CD8+ TILs in *POLE*-mutant and MSI-H ECs than in MSS tumors [8,9]. Analysis of the TCGA EC data set revealed that *POLE*-mutant tumors express higher transcripts of *PD-L1*, *PD-L2*, *CD8A*, and *PD-1* [19]. Talhouk et al. found elevated infiltration by both T cells (CD3+CD8+ TILs and CD3−CD8+ TILs) and B-lineage cells (CD138−CD79a+ and CD138+CD79a+) in *POLE*-mutant and MMRd tumors than in p53-abn and -wt subtypes. The authors also demonstrated a lower level of CD8+ TIL activation and/or exhaustion in p53wt tumors than the other three subtypes [11].

MSI-H and TP53-wt subtypes, accounting for approximately 70% of ECs, display intermediate and indistinguishable prognoses [4,5], highlighting the need for the sub-risk stratification of patients within these two subtypes. Intriguingly, we identified several immunological genes with potential prognostic value within molecular subtypes of EC. High *CCR5* expression was a predictor for longer OS within MSI-H tumors. *CCR5* gene encodes C-C chemokine receptor type 5, a seven-transmembrane G protein-coupled receptor that binds to various ligands. The *CCL5/CCR5* axis promotes tumor progression through a spectrum of mechanisms, such as enhancing invasion and metastasis of tumors, reducing tumor cell resistance to drugs, and recruiting immune and stromal cells [20]. *CCR5* is selectively overexpressed in breast cancer cells and promotes tumor metastases, which is associated with poor prognosis [21]. In colorectal cancer cells, the blockage of *CCR5* suppresses cell proliferation, migration, and clonogenic ability [22]. On the other hand, as a double-edged sword, the *CCL5/CCR5* axis also promotes antitumor immunity by recruiting tumor-infiltrated-T cells and dendritic cells [23,24]. Our study demonstrated *CCR5* as a predictor of favorable prognosis in MSI-H EC for the first time. We also found that the upregulation of *CCR5* was accompanied by elevated intraepithelial CD3+ and CD8+ TILs in MSI-H EC. Our observation is consistent with the previous finding that *CCR5* expression is positively associated with tumor immune cell infiltration [25]. Moreover, Talhouk et al. described a positive association of CD8+CD3+and CD3+CD8− TILs with disease-specific survival(DSS) within MSI-H EC [11]. Collectively, all these observations support the prognostic significance of *CCR5* expression within MSI-H EC.

Our study identified *IL-10* as a potential adverse prognostic factor within *TP53*-wt tumors. *IL-10* gene encodes for interleukin 10, a cytokine that serves as a pleiotropic immunomodulatory factor, predominantly with immunosuppressive effects [26,27]. The increased serum level of interleukin 10 has been described as a predictor of unfavorable outcomes in patients with several cancers (colorectal cancer, renal cell carcinoma, etc.) [28,29,30]. Our study is the first one demonstrating the adverse prognostic effect of *IL-10* gene expression in *TP53*-wt EC. Our results also revealed that the low-*IL10* subgroup (predicted to have a better prognosis) tended towards a TIME landscape of upregulated stromal M1 macrophages compared with the high-*IL10* subgroup. Interestingly, Talhouk et al. showed an association of stromal B cells with increased disease-specific survival within the *TP53*-wt EC [11]. Activated M1 macrophages release pro-inflammatory factors and suppress tumor growth. High infiltration of M1 macrophages has been described in EC patients with a better prognosis [31].

Our study also identified *TNFRSF14* as an independent prognostic factor within the *TP53*-mutant subtype, with higher expression associated with longer OS. *TNFRSF14* encodes for a receptor (HVEM) expressed on T cells, B cells, monocytes, and immature dendritic cells. It functions in lymphocyte activation and regulates the antitumor immune response [32]. *TNFRSF14* induces apoptosis and suppresses proliferation, thus playing a tumor-suppressive role in bladder cancer, which may confer a better prognosis for patients with upregulated *TNFRSF14* expression [33]. In vitro evidence revealed that the inactivation of *TNFRSF14* promotes the migration and invasiveness of EC cells by inducing the EMT [34]. Our results, for the first time, demonstrate the prognostic significance of *TNFRSF14* within the *TP53*-mutant subtype. The prolonged survival of the subgroup expressing higher *TNFRSF14* is likely attributed to the increased intraepithelial CD8+ TILs (Figure 5E), which has been previously associated with a better prognosis in EC patients [35]. However, we also observed more PD-L1+ cells in the high *TNFRSF14* subgroup. The association of PD-L1 expression with prognosis remains elusive in EC patients since relevant studies showed controversial results [36,37]. Our study suggests overexpression of PD-L1 may impact patient outcomes in a heterogeneous manner across varied subtypes of EC, which merits further exploration.

The major limitation of our study is that the follow-up time is not long enough to obtain mature survival data; thus, the prognostic significance of genes identified from the TCGA dataset cannot be validated in our cohort. The differential TIME landscapes between subgroups of our cohort stratified by transcriptome signatures might suggest their prognostic impact. Furthermore, the study enrolled a relatively small number of patients. After molecular classification, the number of patients in each subtype was limited. The finding of our study definitely needs further validation in a larger external cohort with a longer flow-up time.

In conclusion, our study demonstrates that targeted DNA sequencing is an effective one-stop strategy to classify EC patients. Moreover, we comprehensively interrogated the immunomodulatory transcriptome signatures and TIME landscape across four subtypes. Our study identified several immunomodulatory genes that may serve as prognostic predictors to sub-stratify patients within subtypes. Our results reveal the potential of refining the risk stratification in EC using the immunomodulatory signature, which may better guide the clinical management and decision-making for patients with EC.

## 4. Methods and Materials 

### 4.1. Patients and Study Design

Patients with EC were prospectively recruited and underwent surgical staging in Peking Union Medical College Hospital from March 2021 to July 2021 according to the following criteria. Inclusion criteria: (1) newly diagnosed EC histologically confirmed by two independent pathologists; (2) underwent systemic imaging evaluation before surgery; (3) did not receive preoperative chemoradiotherapy or hormonotherapy; (4) provision of tumor surgical specimen measuring >1 mm in diameter. Exclusion criteria: (1) autoimmune diseases; (2) active hepatitis A, B, C, or HIV infection; (3) active syphilis infection. Patients’ clinical and histopathological information were collected. Patients were followed up postoperatively until May 2022. 

**Figure 6:**
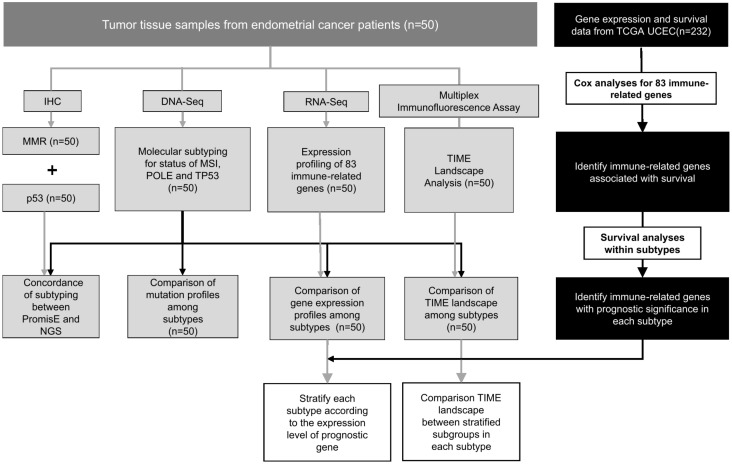
Flowchart of the study design. TCGA-UCEC: the Cancer Genome Atlas Uterine Corpus Endometrial Carcinoma; TIME: Tumor Immune Microenvironment; IHC: immunohistochemical assay; MMR: mismatch repair.

As illustrated in Figure 6, DNA sequencing was performed with 50 formalin-fixed, paraffin-embedded (FFPE) tumor samples for molecular subtyping of patients according to *POLE*, *TP53*, and MSI status. MLH1, PMS2, MSH2, MSH6, and p53 were evaluated using IHC staining. *TP53* and MSI statuses determined from DNA sequencing were compared with corresponding IHC results. RNA sequencing and multiplex immunofluorescence assay were conducted in parallel with the same 50 samples to compare the expression profile of 83 immune-related genes and TIME landscape among different molecular subtypes. Gene expression (https://portal.gdc.cancer.gov/projects/TCGA-UCEC, accessed on 5 June 2021) and survival data (https://xenabrowser.net/datapages/, accessed on 5 June 2021) of TCGA-UCEC were retrieved to screen for prognostic genes. The prognostic value of selected candidates was further investigated within each subtype. Subsequently, patients of each subtype from our cohort were sub-stratified according to the expression of the identified prognostic gene, and the TIME landscape was compared between subgroups. All procedures performed in studies involving human participants were in accordance with the 1964 Helsinki declaration and its later amendments (https://www.wma.net/policies-post/wma-declaration-of-helsinki-ethical-principles-for-medical-research-involving-human-subjects/, accessed on 5 June 2021). The study was approved by the Institutional Ethics Committee at Peking Union Medical College Hospital (No. JS-2884). All patients provided written informed consent.

### 4.2. DNA Sequencing and Molecular Classification of Patients

DNA was extracted from FFPE tumor tissues using the QIAamp DNA FFPE tissue kit (Qiagen, Hilden, Germany), following the manufacturer’s instructions. Capture-based targeted sequencing was performed using a 520-gene panel (OncoScreen^®^Plus, Burning Rock Biotech, Guangzhou, China) as previously described [38,39,40]. Data analyses, including variants calling and interpretation, copy number variation, TMB estimation, and MSI status assessment, were carried out using standardized pipelines based on the methods described previously [40].

Patients were subtyped based on the genomic signatures identified by DNA-sequencing in the order of *POLE* hotspot mutation (P286R, V411L, S297F, A456P, or S459F), MSI-H, and *TP53* mutation. Patients without any of the three signatures were determined as *TP53*-wt.

### 4.3. RNA Sequencing and Gene Expression Profiling

RNA was extracted from FFPE samples using an All prep DNA/RNA FFPE kit (Qiagen, Hilden, Germany). The quantity and quality of extracted RNA were measured using Qubit RNA HS assay (Thermo Fisher Scientific, Waltham, MA, USA) and LabChip GXII touch 24 (Perkin Elmer, Waltham, MA, USA), respectively. RNA sample with a concentration ≥ 4ng/uL and DV200 ≥ 40% was considered eligible for subsequent library construction. After strand-specific cDNA synthesis, dA-tailing, unique molecular identifier adaptor ligation, and PCR amplification, the products were hybridized with the capture probe of the OncoRNA panel (Burning Rock Biotech, Guangzhou, China). The panel consists of 83 immunomodulatory genes (Table S1). The prepared libraries were sequenced, and the sequencing data were processed as previously described [41]. Gene-level expression values were computed as transcripts per million (TPM) and normalized to Z-scores before clustering. A heatmap of gene expression was drawn using Pheatmap (1.0.12). Genes were categorized into different gene sets designated as signatures of T-effector and IFN-γ, IPRES_eCD8T, EMT, and TGF-Beta (Table S1), according to previous publications [17,42,43]. The ssGSVA program [44] was used to calculate a single-sample gene set enrichment score for each signature. A total of 50 samples had been finally analyzed.

### 4.4. Multiplex Immunofluorescence Assay

FFPE blocks were serially sliced into sections of 5 μm and subjected to multiplex immunofluorescence staining using the PANO 7-plex IHC kit (Panovue, Beijing, China) according to the manufacturer’s instruction. Two panels were analyzed in this study and stained with different combinations of primary antibodies: one was stained with antibodies for PD-L1, PD-1, CD3, CD8, and Pan-CK and the other was stained with antibodies for CD68, CD163, CD56, and Pan-CK. Subsequently, slides were incubated with horseradish peroxidase (HRP)-conjugated secondary antibody followed by tyramide signal amplification using TSA Fluorescence Kits (Panovue, Beijing, China).

The Mantra System (PerkinElmer, Waltham, MA, USA) was used to scan the slides. The fluorescent spectra were set at 20-nm wavelength intervals from 420 to 720 nm. A spectral library was established using the extracted images and used for multispectral unmixing by inForm image analysis software (PerkinElmer, Waltham, MA, USA). The present study reported the densities and positive rates of PD1+, PD-L1+, CD3+ (designating T cells), CD8+ (designating cytotoxic T cells), CD56+ (designating for NK cells), CD163−CD68+ (designating M1 macrophages), CD163+CD68+ (designating M2 macrophages) markers on cells in both the epithelial and stromal compartments of tumors.

### 4.5. Statistical Analyses

All statistical analyses were performed using R software (version 3.5.3). Differences among groups were compared using the Chi-square test or Fisher’s exact test for categorical variables and the Kruskal–Wallis test for continuous variables. The Wilcoxon test was used to compare to differences between groups. Multivariate Cox regression analysis was performed to adjust for confounders. Kaplan –Meier analysis was used to estimate survival, and a log-rank test was used to determine the differences in the multiple survival metrics between groups. Statistical significance was defined as *p* < 0.05.

## Figures and Tables

**Table 1:** Characteristics of patients.

	Overall (*n* = 50)	MSI-H Subgroup (*n* = 10)	*POLE*-Mutant Subgroup (*n* = 5)	*TP53*-Mutant Subgroup (*n* = 9)	*TP53*-wt Subgroup (*n* = 26)	*p*
Age						0.038 ^c^
Median [IQR]	57.0 (52.0, 66.0]	61.0 (57.5, 67.5]	54.0 (47.0, 55.0]	67.0 (58.0, 71.0]	56.0 (46.8, 62.3]	
FIGO stage, *n* (%)						0.961 ^a^
IA	24 (48.0)	4 (40.0)	4 (80.0)	3 (33.3)	13 (50.0)	
IB	6 (12.0)	2 (20.0)	0 (0.0)	1 (11.1)	3 (11.5)	
II	1 (2.0)	0 (0.0)	0 (0.0)	0 (0.0)	1 (3.9)	
IIIA	4 (8.0)	1 (10.0)	0 (0.0)	1 (11.1)	2 (7.7)	
IIIC1	6 (12.0)	1 (10.0)	1 (20.0)	1 (11.1)	3 (11.5)	
IIIC2	5 (10.0)	2 (20.0)	0 (0.0)	2 (22.2)	1 (3.9)	
IVB	4 (8.0)	0 (0.0)	0 (0.0)	1 (11.1)	3 (11.5)	
Histologic Type, *n* (%)						<0.001 ^b^
Endometrioid	43 (86.0)	10 (100.0)	5 (100.0)	2 (22.2)	26 (100.0)	
Serous	3 (6.0)	0 (0.0)	0 (0.0)	3 (33.3)	0 (0.0)	
Clear cell	4 (8.0)	0 (0.0)	0 (0.0)	4 (44.44%)	0 (0.0)	
Histologic Grade, *n* (%)						0.021 ^a^
G1	14 (28.0)	4 (40.0)	2 (40.0)	0 (0.0)	8 (30.8)	
G2	21 (42.0)	4 (40.0)	1 (20.0)	2 (22.2)	14 (53.9)	
G3	15 (30.0)	2 (20.0)	2 (40.0)	7 (77.8)	4 (15.4)	
LVSI, *n* (%)						0.048 ^b^
No	29 (58.0)	7 (70.0)	0 (0.0)	6 (66.7)	16 (61.5)	
Yes	21 (42.0)	3 (30.0)	5 (100.0)	3 (33.3)	10 (38.5)	
Myometrial invasion, *n* (%)						0.078 ^a^
No	4 (8.0)	3 (30.0)	0 (0.0)	0 (0.0)	1 (3.9)	
<50%	27 (54.0)	3 (30.0)	4 (80.0)	4 (44.4)	16 (61.5)	
≥50%	19 (38.0)	4 (40.0)	1 (20.0)	5 (55.6)	9 (34.6)	
Lymph node metastasis, *n* (%)						0.955 ^b^
No	37 (74.0)	7 (70.0)	4 (80.0)	6 (66.7)	20 (76.9)	
Yes	12 (24.0)	3 (30.0)	1 (20.0)	2 (22.2)	6 (23.1)	
ND	1 (2.0)	0 (0.0)	0 (0.0)	1 (11.1)	0 (0.0)	
ER, *n* (%)						0.006 ^a^
Negative	10 (20.0)	1 (10.0)	1 (20.0)	6 (66.7)	2 (7.7)	
Positive (>95%)	28 (56.0)	5 (50.0)	3 (60.0)	1 (11.1)	19 (73.1)	
Partial positive (≤95%)	12 (24.0)	4 (40.0)	1 (20.0)	2 (22.2)	5 (19.2)	
PR, *n* (%)						0.002 ^a^
Negative	9 (18.0)	1 (10.0)	1 (20.0)	6 (66.7)	1 (3.9)	
Positive (>95%)	28 (56.0)	5 (50.0)	3 (60.0)	1 (11.1)	19 (73.1)	
Partial positive (≤95%)	12 (24.0)	4 (40.0)	1 (20.0)	2 (22.2)	5 (19.2)	
ND	1 (2.0)	0 (0.0)	0 (0.0)	0 (0.0)	1 (3.9)	
Ascitic fluid Cytology, *n* (%)						0.395 ^b^
Negative	40 (80.0)	7 (70.0)	5 (100.0)	5 (55.6)	23 (88.5)	
Positive	5 (10.0)	0 (0.0)	0 (0.0)	2 (22.2)	3 (11.5)	
ND	5 (10.0)	3 (30.0)	0 (0.0)	2 (22.2)	0 (0.0)	
TMB, mutations/Mb						<0.001 ^c^
Median [IQR]	7.5 (4.8,26.8]	31.4 (22.6,38.9]	325.4 (295.2,332.5]	5.6 (4.8,7.9]	5.96 (4.8,7.8]	
Ki67 expression, %						0.001 ^c^
Median [IQR]	70.0 (40.0,80.0]	72.5 (70.0,80.0]	70.0 (60.0,80.0]	80.0 (70.0,80.0]	50.0 (20.0,68.8]	
ND, *n* (%)	3 (6.00)	1 (10.00)	0 (0.00)	0 (0.00)	2 (7.69)	

Note: *p* value was calculated by Chi-square test (a), Fisher’s exact test (b) or Kruskal–Wallis test (c). Abbreviations: LVSI: lymphovascular space invasion; ER: estrogen receptor; PR: progesterone receptor; TMB: tumor mutational burden; SD: standard deviation; IQR: interquartile range; ND, no data.

## Data Availability

The datasets generated and/or analysed during the current study are available in the NGDC repository [https://ngdc.cncb.ac.cn/, OMIX002704, OMIX002705].

## Supplementary Materials

The supporting information can be downloaded at: https://www.mdpi.com/article/10.3390/ijms24021791/s1.
