# Supplementary figures and images for: Immunologic Signatures across Molecular Subtypes and Potential Biomarkers for Sub-Stratification in Endometrial Cancer
Source: Int J Mol Sci. 2023 Jan 16;24(2):1791. doi: 10.3390/ijms24021791 (PMC9861911; doi:10.3390/ijms24021791)

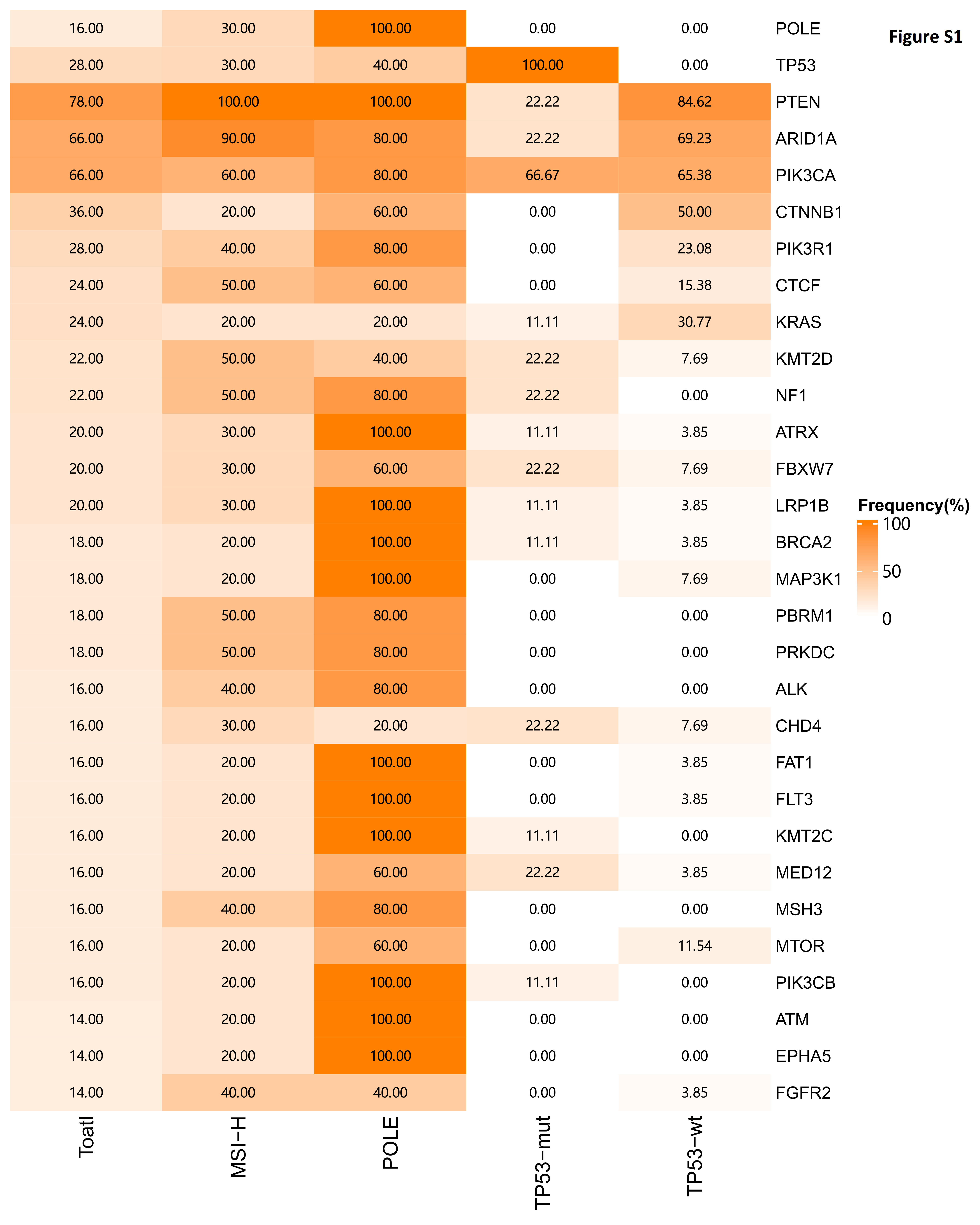

Supplement: Supplementary file 1 [file ijms-24-01791-s001.zip › Figure S1.png]

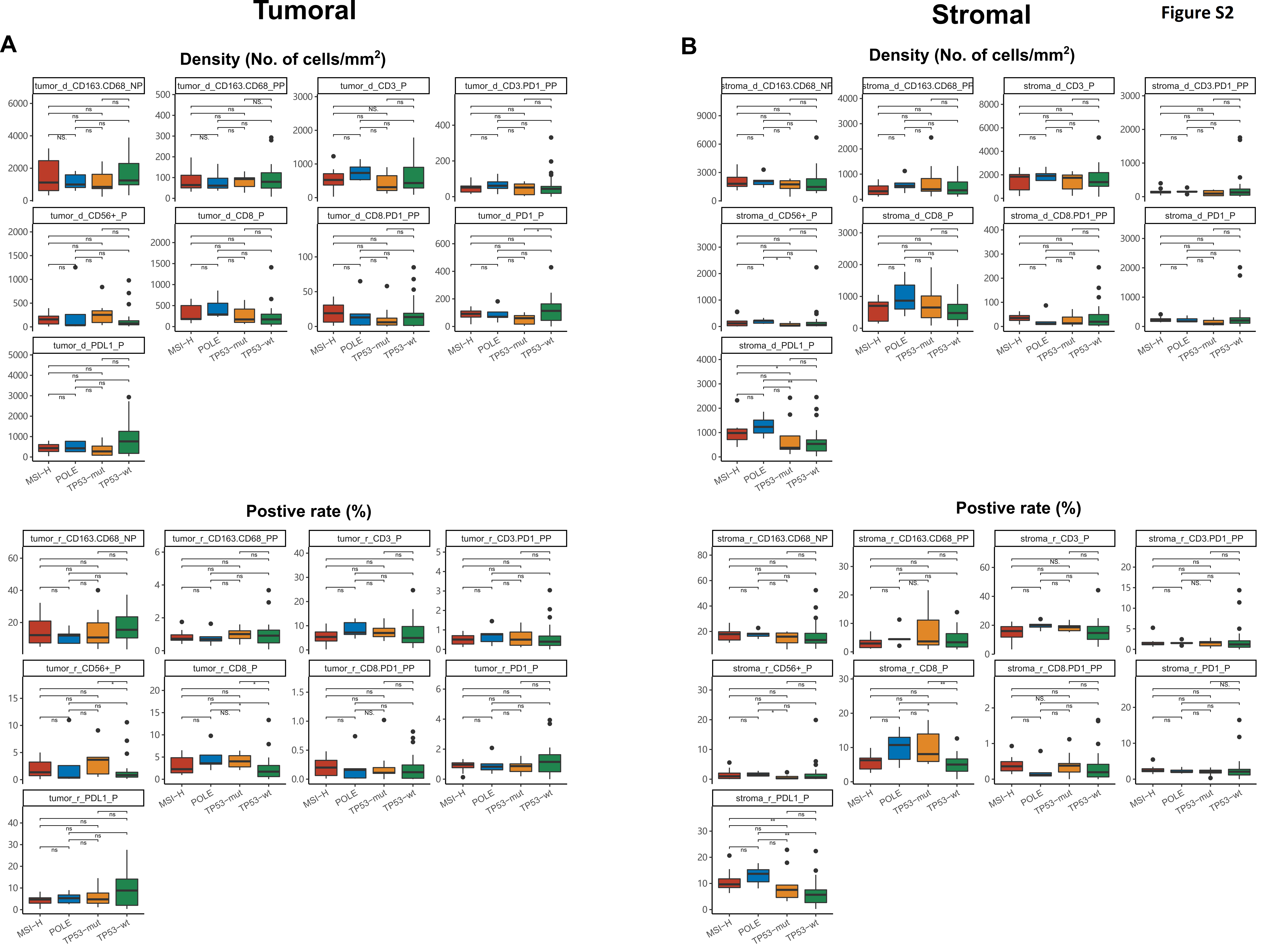

Supplement: Supplementary file 1 [file ijms-24-01791-s001.zip › Figure S2.png]
